# Metabolic regulation of gut barrier integrity in IBD: protein, lipid, and carbohydrate pathways

**DOI:** 10.3389/fnut.2026.1873821

**Published:** 2026-09-07

**Authors:** Ying Wang, Yinying Wang, Yuanjie Fu, Junxin Li, Tiangang Li, Liyun Song, Yanghuan Ou, Li Li, Yueying Wu, Jinyuan Yan, Zhongshan Yang

**Affiliations:** 1 Yunnan Provincial Key Laboratory of Integrated Traditional Chinese and Western Medicine for Chronic Disease in Prevention and Treatment, Yunnan University of Traditional Chinese Medicine, Kunming, Yunnan, China; 2 Center Laboratory of the Second Affiliated Hospital, Kunming Medical University, Kunming, China; 3 Yunnan Provincial Department of Education, Engineering Research Center of Classic Formula Regulate Immunity in Chronic Disease Prevention and Treatment, Kunming, Yunnan, China

**Keywords:** nutrients, inflammatory bowel disease, intestinal barrier, metabolism, microbiota, immunity

## Abstract

Dietary macronutrients—proteins, lipids, and carbohydrates—influence the pathogenesis of inflammatory bowel disease (IBD) through distinct but interconnected metabolic pathways that extend beyond their roles as energy substrates. This narrative review synthesizes current evidence on how each macronutrient class modulates gut microbiota composition, intestinal barrier integrity, and mucosal immune responses. In the protein domain, the study examines how amino acid metabolism, particularly the tryptophan–kynurenine–indole axis and the arginine–inducible nitric oxide synthase–arginase balance, serves as a regulatory interface between microbial signals and immune cell function, and evaluates the clinical potential of targeted amino acid supplementation. In the lipid domain, a multi-level cascade is traced from phospholipid-dependent barrier structure through the pro-inflammatory/pro-resolving lipid mediator network to immune cell lipid reprogramming, and the study discusses how systemic lipoprotein abnormalities both reflect and amplify intestinal inflammation. In the carbohydrate domain, the study distinguishes between fermentable fiber and high-glycemic carbohydrates, and presents a dual-pathway model in which excess sugar drives IBD through two parallel routes: a microbiota-dependent pathway involving mucus depletion, barrier breach, and short-chain fatty acid deprivation; and a microbiota-independent pathway that directly impairs the metabolism of colonic epithelial stem cells. Throughout the study the emphasis is on the bidirectional nature of diet–inflammation interactions and identifies convergent mechanisms across macronutrient classes. The study concludes by outlining the translational challenges that must be addressed—cross-nutrient interactions, causal inference, and disease-stage-specific interventions—to move from generic dietary guidance toward mechanism-informed, personalized nutritional strategies for IBD, and highlights the need for well-designed intervention studies that test these strategies in defined patient subsets.

## Graphical abstract

**Figure:**
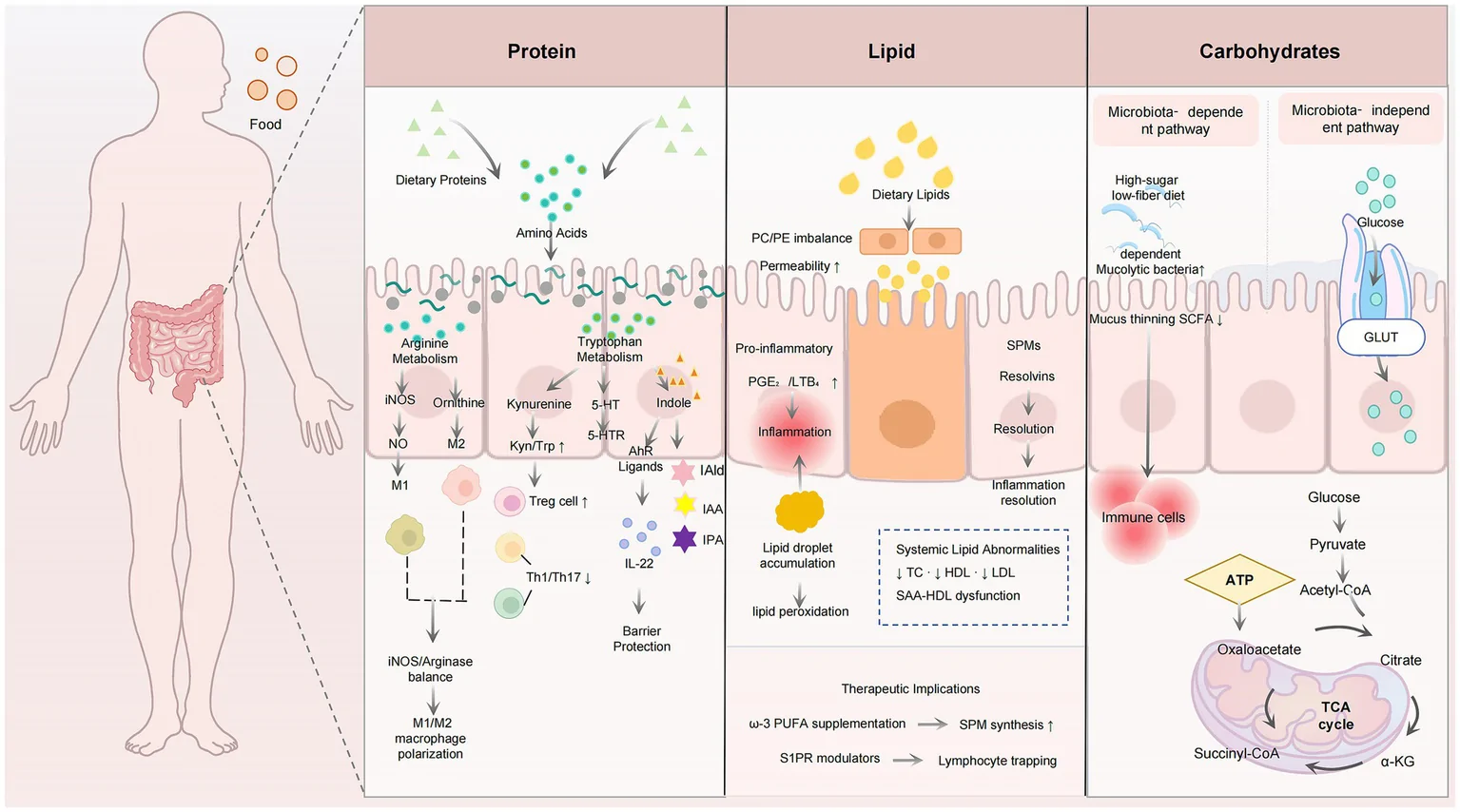
Dietary macronutrients—proteins, lipids, and carbohydrates—regulate intestinal homeostasis through distinct but interconnected metabolic axes involving the gut microbiota, epithelial barrier, and immune system. Protein-derived amino acids, particularly arginine and tryptophan, are metabolized through parallel pathways. Arginine metabolism via the inducible nitricoxide synthase–arginase balance modulates M1/M2 macrophage polarization, influencing the balance between inflammation and tissue repair. Tryptophan is processed through three parallel branches: the host-derived kynurenine pathway, which regulates Treg/Th1/Th17 balance; the host-derived serotonin (5-HT) pathway, which signals through 5-HT receptors on immune cells; and the microbiota-derived indole pathway, which produces aryl hydrocarbon receptor (AhR) ligands and induces IL-22 for epithelial barrier protection. Lipid metabolism influences intestinal inflammation through a network of pro-inflammatory mediators (PGE₂/LTB₄) and specialized pro-resolving mediators (SPMs), with dysregulated lipid metabolism driving lipid droplet accumulation, lipid peroxidation, while systemic lipid abnormalities (reduced TC, HDL, LDL, and SAA-HDL dysfunction) reflect inflammation-driven remodeling. Excessive carbohydrate intake, particularly high-sugar, low-fiber diets, drives a dual-pathway model of mucosal injury: microbiota-dependent mechanisms involving mucolytic bacterial expansion and short-chain fatty acid depletion, and microbiota-independent mechanisms involving GLUT(glucose transporters)-mediated glucose uptake and glycolysis–tricarboxylic acid cycle uncoupling, leading to adenosine triphosphate deficiency and impaired epithelial stem cell proliferation. Together, these macronutrient-driven pathways converge on immune cell activation, intestinal barrier disruption, and chronic inflammation, highlighting potential therapeutic targets including *ω*-3 polyunsaturated fatty acids (PUFA) supplementation, SPM synthesis enhancement, and S1PR modulators.

## 1 Introduction

Inflammatory bowel disease (IBD), encompassing Crohn’s disease (CD) and ulcerative colitis (UC), is a chronic, relapsing inflammatory condition of the gastrointestinal tract whose global incidence continues to rise (1).

In the largest multi-ancestry meta-analysis to date, combining East Asian and European populations, over 320 susceptibility loci for IBD have now been confirmed (2), and this figure is expected to continue growing as larger multi-ancestry studies and rare-variant analyses emerge. However, these genetic variants explain only a modest proportion of heritability, a finding that has driven a paradigm shift from purely genetic determinism toward gene–environment interactions, in which diet—as a modifiable environmental factor—has emerged as a key research focus.

The relationship between dietary macronutrients and IBD is both profound and bidirectional. Dietary proteins, lipids, and carbohydrates do not merely supply calories; their metabolicprocessing by host enzymes and the gut microbiota generates bioactive molecules that directly regulate epithelial integrity, immune cell polarization, and the composition of the microbial ecosystem itself. Conversely, the chronic inflammatory state of IBD reshapes host metabolism, altering amino acid profiles, systemic lipid levels, and energy homeostasis in ways that can compound nutritional deficits and perpetuate inflammation.

Beyond macronutrient metabolism per se, IBD is frequently complicated by protein–energy malnutrition, sarcopenia, and deficiencies of specific micronutrients. The global prevalence of malnutrition in IBD ranges from 20 to 85% (3), depending on disease activity and the assessment tool used, while sarcopenia—defined as low muscle mass and function—affects approximately 25–60% of patients with IBD and is independently associated with increased risk of surgical complications, hospitalization, and mortality (4). Deficiencies of iron, zinc, selenium, and vitamins B₁₂ and D are also common, resulting from multifactorial causes including reduced dietary intake, intestinal malabsorption, chronic blood loss, and medication-related effects (5). These nutritional derangements are not merely passive consequences of intestinal inflammation but actively perpetuate disease by impairing immune function, delaying mucosal healing, and diminishing quality of life. This broader context of nutritional deterioration underscores the importance of integrated management strategies that address both macronutrient and micronutrient needs, and provides the clinical rationale for the mechanistic pathways discussed in the sections that follow.

Among dietary proteins, both total intake and protein source influence colitis susceptibility. Protein fermentation in the colon releases metabolites—hydrogen sulfide (H_2_S), ammonia, phenolic compounds—that compromise barrier function at high concentrations, while the amino acid composition of different protein sources differentially shapes the gut microbiota. Beyond their structural role, specific amino acids function as critical metabolic regulators: tryptophan metabolism through the kynurenine, serotonin, and indole pathways integrate microbial and immune signals to control barrier repair and inflammation resolution, while the arginine–iNOS–arginase axis determines whether the local immune milieu tilts toward tissue injury or repair. These mechanistic insights have prompted clinical interest in amino acid supplementation, though robust evidence for efficacy in human IBD remains limited.

Dietary lipids influence IBD through multiple interconnected levels. The phospholipid composition of the enterocyte membrane itself is altered in IBD, shifting the precursor pool toward pro-inflammatory eicosanoid production at the expense of specialized pro-resolving mediators (SPMs). The sphingolipid network—particularly the ceramide/S1P balance—adds a further dimension to this mediator landscape. Within the inflamed mucosa, immune cells undergo lipid metabolic reprogramming, with macrophages shifting toward fatty acid synthesis and lipid droplet accumulation that actively sustains inflammation. Systemically, IBD patients exhibit a characteristic but paradoxical lipid profile—reduced total, HDL, and LDL cholesterol—that reflects a complex interplay between inflammation-driven lipoprotein remodeling, malnutrition, and potential immunoregulatory dysfunction.

For carbohydrates, the critical distinction lies not in total quantity but in carbohydrate type. High-glycemic carbohydrates and fiber-poor diets promote IBD through two parallel routes: a microbiota-dependent pathway in which mucolytic bacterial expansion, barrier erosion, and SCFA depletion drive chronic immune activation, and a microbiota-independent pathway in which excess luminal glucose directly impairs colonic stem cell metabolism, creating an energy deficit that compromises epithelial repair. Epidemiological evidence supports this dual-pathway model, with sugar intake—but not total carbohydrate consumption—consistently associated with increased IBD risk.

This review systematically examines how dietary proteins, lipids, and carbohydrates each modulate IBD pathogenesis through their respective metabolic routes. For each macronutrient class, the cascade is traced from dietary intake through metabolic processing to functional effects on the gut barrier, the microbiota, and the mucosal immune system, highlighting areas of mechanistic convergence and persistent knowledge gaps. Then the study considers the translational implications of these findings, focusing on the challenges of cross-nutrient interactions, causal inference, and the need for disease-stage-specific nutritional strategies. The aim of the study is to provide a synthesis that moves beyond generic dietary recommendations and toward a mechanism-informed framework capable of guiding the next generation of precision nutrition studies in IBD.

This narrative review was based on a comprehensive literature search in PubMed and Web of Science up to February 2026. High-quality mechanistic studies prioritized meta-analyses, systematic reviews, and key clinical trials, and organized the evidence thematically according to macronutrient class (protein, lipid, and carbohydrate) and the level of biological organization (gut microbiota, epithelial barrier, and mucosal immunity). Based on the extensive study of this topic and the mechanistic focus of this review, a narrative synthesis approach was adopted rather than a formal systematic review methodology. Relevant references were also identified through cross-referencing of primary articles and reviews.

## 2 Protein and IBD

### 2.1 Physiologic basis of protein metabolism and IBD association

Dietary protein that escapes small-intestinal digestion reaches the colon, where it undergoes fermentation by the gut microbiota. This process generates a range of metabolites with well-documented effects on the colonic epithelium, including H₂S, ammonia, phenolic compounds, and amines—all of which are elevated in the feces of patients with IBD (6). Through these and other mechanisms, fermentation products can compromise epithelial barrier integrity and promote mucosal inflammatory cascades (7, 8).

However, protein fermentation also yields metabolites with potentially beneficial functions. Branched-chain fatty acids (BCFAs)—namely isobutyrate, 2-methylbutyrate, and isovalerate, derived from valine, leucine, and isoleucine, respectively—have not been associated with adverse effects and may serve as energy substrates for colonocytes (9). In addition, indolic compounds produced from microbial tryptophan metabolism activate the aryl hydrocarbon receptor (AhR) on host cells, thereby enhancing barrier function and attenuating intestinal inflammation via IL-22 induction (10). Consequently, the net impact of protein fermentation on gut health hinges on the balance between detrimental and beneficial metabolite production—a balance shaped by protein source, gut microbial composition, and the host metabolic environment.

Consistent with these observations, when the relative contribution of different macronutrients to colitis susceptibility was systematically compared, dietary protein content emerged as the dominant dietary driver of intestinal permeability and colitis severity (11). Beyond total intake, however, the source of dietary protein profoundly influences the intestinal microenvironment. Notably, colitis exacerbation has been predominantly linked to specific animal protein sources rather than to animal protein as a homogeneous category. Meta-analytical evidence indicates that red meat consumption is significantly associated with an increased risk of UC, with each additional 100 g/day raising incidence risk by 65%; in contrast, fish protein has not shown a consistent association with IBD risk (12).

This differentiation—with red meat conferring risk, fish showing no consistent association—underscores the importance of distinguishing protein sources rather than treating all animal proteins as equivalent.

From a microbiological perspective, animal-protein-rich diets tend to enrich mucolytic bacteria (13, 14). It is important to note, however, that not all mucolytic bacteria are intrinsically detrimental. The role of *Akkermansia muciniphila* in intestinal health is highly context-dependent. Under healthy conditions, its controlled mucolytic activity, coupled with mucosal regeneration, contributes to normal turnover of the intestinal barrier and immune regulation, thereby supporting homeostasis. In contrast, under pathological states—such as severe intestinal inflammation or when the mucus layer is already compromised—excessive mucolytic activity may disrupt barrier integrity and exacerbate mucosal injury (15). This dual nature explains both the depletion of *A. muciniphila* observed in IBD patients and the protective effects of exogenous supplementation in experimental colitis models (16–19). By contrast, species belonging to the genera *Enterococcus* and *Streptococcus* are more consistently implicated in IBD and are associated with a clearer pro-inflammatory potential (13, 14). Conversely, plant-based protein diets appear to favor beneficial taxa such as *Lactobacillus* and *Bifidobacterium* while suppressing inflammatory signaling pathways (20). These microbial shifts may contribute to the differential effects of animal- versus plant-derived proteins on intestinal homeostasis, further highlighting that the relationship between dietary protein and IBD is mediated not only by metabolite production but also by the specific compositional changes induced in the gut microbiota.

### 2.2 Amino acid metabolism as a regulatory Nexus in IBD

The metabolic fate of dietary amino acids extends far beyond their role as protein building blocks. In the context of IBD, amino acid metabolism emerges as a critical regulatory layer that integrates microbial signals, epithelial barrier function, and immune cell responses. Profiling studies have revealed a distinct plasma amino acid signature in IBD patients, characterized by reduced concentrations of histidine, threonine, and tryptophan, among others, while certain amino acids show disease-specific alterations (21). These systemic changes reflect the combined effects of dietary protein intake, intestinal malabsorption, and the metabolic demands of chronic inflammation. Two amino acid pathways—arginine and tryptophan metabolism—stand out for their centrality in orchestrating immune homeostasis and mucosal integrity in the inflamed gut.

### 2.3 Arginine metabolism: a metabolic checkpoint for inflammation and repair

Alterations in arginine metabolism have been consistently documented in IBD, with active colitis associated with reduced colonic arginine levels and disrupted metabolic flux through its principal routes. Arginine is metabolized via four pathways: nitric oxide (NO) synthesis by inducible nitric oxide synthase (iNOS), conversion to ornithine and urea by arginase, agmatine production via arginine decarboxylase, and creatine synthesis via arginine: glycine amidinotransferase. Among these, the iNOS–arginase balance serves as a metabolic checkpoint that profoundly influences the inflammatory versus reparative phenotype of the intestinal mucosa.

Cellular arginine availability is governed by members of the solute carrier family 7 (SLC7) of cationic amino acid transporters. SLC7A1 (CAT-1) supplies arginine constitutively for basal cellular metabolism (22), whereas SLC7A2 (CAT-2) is induced during macrophage activation and plays a dynamic role in regulating inflammation (23). In the inflamed intestine of UC patients, SLC7A2 expression is decreased, contributing to reduced arginine uptake and perturbed local metabolism; accordingly, Slc7a2-deficient mice exhibit heightened susceptibility to dextran sulfate-induced colitis (24), underscoring the functional importance of arginine transport in maintaining intestinal homeostasis.

Once inside the cell, arginine is directed toward two competing enzymatic branches with opposing functional outcomes. Arginase 1 (ARG1), a cytosolic enzyme predominantly expressed in myeloid cells, is upregulated in the inflamed mucosa in an IL-4/IL-13- and microbiota-dependent manner, and its activity correlates with the degree of inflammation in IBD patients. Functional studies have shown that ARG1 impedes colitis resolution by altering the gut microbiome and metabolome (25). In contrast, arginase 2 (ARG2), a mitochondrial isoform broadly distributed in extrahepatic tissues, plays a protective role in the intestinal epithelium by supporting endogenous spermidine synthesis, and its activity exerts palliative effects in UC (26). This isoform-specific divergence—pro-inflammatory ARG1 versus protective ARG2—illustrates the complexity of arginine metabolism and cautions against viewing arginase as a unitary pathway.

The ornithine generated by arginase feeds into polyamine biosynthesis, with ornithine decarboxylase (ODC1) as the rate-limiting enzyme (27). Polyamines (putrescine, spermidine, and spermine) are essential for cell viability, proliferation, and differentiation, and play a critical role in mucosal repair. In the intestinal epithelium, ODC1 also supports group 3 innate lymphoid cell (ILC3) responses through positive regulation of IL-22 transcription, and its deficiency increases susceptibility to enteric infection. Concurrently, in the inflamed mucosa, pro-inflammatory cytokines upregulate iNOS, driving sustained NO production. While NO confers antimicrobial defense, excessive NO generation causes epithelial injury and perpetuates inflammatory cascades. Thus, the metabolic decision between iNOS-mediated NO production and arginase-mediated ornithine synthesis shapes the local environment toward either cytotoxic inflammation or tissue repair. The importance of this balance is further underscored by the observation that macrophage polarization—M1 relying on glycolysis and iNOS for bactericidal activity, M2 engaging oxidative phosphorylation and arginase for tissue remodeling—is intrinsically coupled to arginine metabolism (23).

### 2.4 Tryptophan metabolism: a multi-layered host–microbial regulatory hub

Tryptophan (Trp) metabolism represents the best-characterized amino acid regulatory hub in IBD. Trp is processed through three major enzymatic routes: the kynurenine (Kyn) pathway, the serotonin (5-hydroxytryptamine, 5-HT) pathway, and the indole pathway.

The Kyn pathway accounts for approximately 95% of dietary Trp catabolism and is initiated by the rate-limiting enzyme indoleamine-2,3-dioxygenase 1 (IDO1). IDO1 expression is minimal in normal intestinal tissue but is strongly induced by IFN-*γ*, IL-2, and TNF-*α* (28), linking its activity directly to the inflammatory milieu. In IBD patients, plasma Trp levels are consistently reduced while Kyn levels are elevated, producing an increased Kyn/Trp ratio that correlates with disease activity (21). IDO1-deficient mice are more susceptible to experimental colitis, confirming that IDO1 functions as a non-redundant negative regulator of intestinal inflammation (29). Mechanistically, IDO1-mediated Trp depletion and Kyn production suppress effector T cell responses while promoting regulatory T cell differentiation, thereby restraining excessive inflammation.

The 5-HT pathway produces serotonin, a neurotransmitter with potent immunomodulatory functions in the gut. Enterochromaffin cells (ECs) are the primary source of intestinal 5-HT, releasing it in response to mechanical and chemical luminal stimuli. Elevated 5-HT levels are found in the mucosa of both CD and UC patients, correlating with increased EC density (30–32). 5-HT signals through a family of receptors expressed on dendritic cells, macrophages, lymphocytes, and intestinal epithelial cells (33–36), modulating both innate and adaptive immune responses. Notably, the gut microbiota actively participates in 5-HT regulation: bacterial metabolites, including LPS and cholera toxin, potentiate 5-HT release from ECs (37, 38), while 5-HT itself can influence the growth of specific bacterial taxa (39, 40). However, the functional complexity arising from multiple 5-HT receptor subtypes with opposing effects has hindered a unified understanding of 5-HT’s role in intestinal inflammation. Given the functional complexity arising from multiple 5-HT receptor subtypes with opposing effects, the major receptor-specific functions in the intestinal mucosa are summarized in Table 1.

**Table 1:** Major 5-HT receptor subtypes and their functions in intestinal immunity and epithelial homeostasis.

Receptor	Expression	Key functions in the gut	References
5-HT_1_A	Enteric neurons, epithelial cells, immune cells	Receptor activation delays and mitigates colitis severity; counteracts increased colonic 5-HT content	(137)
5-HT₂A	Macrophages, dendritic cells, smooth muscle, enteric neurons	Mediates pro-inflammatory effects in TNBS-induced colitis	(137)
5-HT₃	Enteric neurons, lymphocytes	Ligand-gated ion channel (unique among 5-HT receptors); regulates peristalsis and immune responses; antagonists ameliorate intestinal inflammation	(138)
5-HT₄	Enteric neurons, epithelial cells, dendritic cells	Promotes mucus secretion, improves barrier function, enhances epithelial wound healing; protects enteric neuronal survival; anti-inflammatory actions	(139)
5-HT₇	T lymphocytes, myeloid cells, intestinal epithelial cells, dendritic cells, macrophages	Regulates plasmacytoid dendritic cell (pDC) homing and IgA^+^ B cell differentiation; promotes experimental colitis	(140)

The third pathway, the microbial indole pathway, converts Trp into AhR ligands. AhR is a ligand-activated transcription factor whose activation in intestinal epithelial cells and immune cells induces IL-22 production, a cytokine essential for epithelial barrier maintenance and antimicrobial defense (41). In Card9-deficient mice, which harbor altered gut microbiota with impaired Trp-to-indole metabolic capacity, AhR activation is defective, IL-22 signaling is compromised, and colitis susceptibility is markedly increased. Fecal microbiota transplantation ameliorates experimental colitis in part by restoring AhR ligand production and downstream IL-22 and IL-10 induction (42–45). These findings establish the Trp-microbiota-AhR-IL-22 axis as a central pathway coupling nutrient metabolism to mucosal immune homeostasis.

### 2.5 Translational perspectives

The mechanistic understanding of amino acid metabolism has spurred multiple therapeutic strategies targeting these pathways. Postbiotic approaches have emerged as a means to deliver the benefits of microbial Trp metabolism without live bacterial administration; for example, postbiotics derived from wheat bran arabinoxylan and *Limosilactobacillus reuteri* remodel gut microbiota and enhance AhR ligands, including indole derivatives, thereby augmenting anti-colitis efficacy through microbiota-dependent AhR signaling (46). Specific indole derivatives have been identified as key mediators of AhR-dependent protection: indole-3-acetic acid (IAA) enhances intestinal mucin sulfation via the AhR–Papss2–Slc35b3 pathway (47), while indole-3-aldehyde (IAld) alleviates intestinal inflammation by activating AhR, inhibiting ROS production and NLRP3 inflammasome activation, and maintaining mitochondrial membrane potential in intestinal epithelial cells (48); IAld also restores high-fat diet-induced intestinal barrier disruption by promoting AhR-mediated intestinal stem cell proliferation (49). While these strategies show considerable preclinical promise, their clinical applicability remains to be established through rigorous human studies. Ultimately, the translation of these mechanistic insights into clinical practice requires further validation in human cohorts and the development of robust biomarkers that capture individual metabolic phenotypes.

Collectively, amino acid metabolism—particularly the arginine–NO and tryptophan–Kyn/5-HT/indole axes—serves as a critical interface between dietary nutrients and the immune–microbial networks that govern intestinal inflammation. Targeting these pathways, whether through supplementation of specific amino acids, modulation of key metabolic enzymes, or microbiota-based interventions, holds considerable therapeutic promise for IBD.

### 2.6 Clinical potential of amino acid supplementation

A substantial proportion of IBD patients exhibit inadequate protein intake, independent of disease activity or phenotype (50). In response, ESPEN guidelines now recommend increasing protein intake to 1.2–1.5 g/kg/day in adults with active disease (51), exceeding the general population recommendation of 1 g/kg/day. Beyond simply meeting total protein requirements, a more refined question has emerged: can supplementation with specific amino acids exert pharmacologic effects that directly modulate intestinal inflammation and barrier function?

Glutamine has received the most attention in this context, and for good reason—it serves as the primary respiratory fuel for intestinal epithelial cells and its concentration is significantly reduced in the colonic mucosa of IBD patients (52). In experimental models, glutamine supplementation attenuates oxidative stress, strengthens tight junction protein expression, and reduces pro-inflammatory cytokine production (53). Notably, rectal glutamine prevented colonic fibrosis in a rat TNBS colitis model, directly addressing a major clinical complication of CD (54). Translating these findings to patients, a glutamine-rich polymeric diet improved outcomes in a randomized controlled trial in children with active CD (55), and ex vivo studies using human intestinal mucosa confirmed that glutamine suppresses IL-1β, IL-6, IL-8, and TNF-*α* production via a post-transcriptional mechanism (56). Despite this preclinical and early clinical promise, the therapeutic effect of glutamine supplementation in large-scale IBD trials has not been robustly established, and questions remain about optimal dosing, route of administration, and which patient subgroups are most likely to benefit.

Beyond glutamine, a diverse array of amino acids has shown protective effects in experimental colitis, although the evidence remains largely preclinical. These amino acids appear to converge on several common mechanistic themes: (i) barrier support and mucin synthesis—threonine, serine, and cysteine promote mucin production and secretion, reinforcing the protective mucus layer (57); (ii) oxidative stress defense—cysteine serves as the rate-limiting substrate for glutathione synthesis, the major intracellular antioxidant (58); (iii) immune regulation—branched-chain amino acids (BCAAs; leucine, valine, isoleucine) are required for innate immune cell function, and their deficiency compromises lymphocyte populations (59), while histidine suppresses macrophage-derived pro-inflammatory cytokines through histamine production (60); and (iv) mucosal repair—L-arginine supplementation reduces colonic inflammation and promotes tissue repair in both TNBS- and DSS-induced colitis (61–63), consistent with its role as a substrate for polyamine synthesis and NO production discussed in Section 2.3. Glycine similarly protects against chemical-induced colitis by inhibiting inflammatory cytokine and chemokine induction (64).

**Figure 1:**
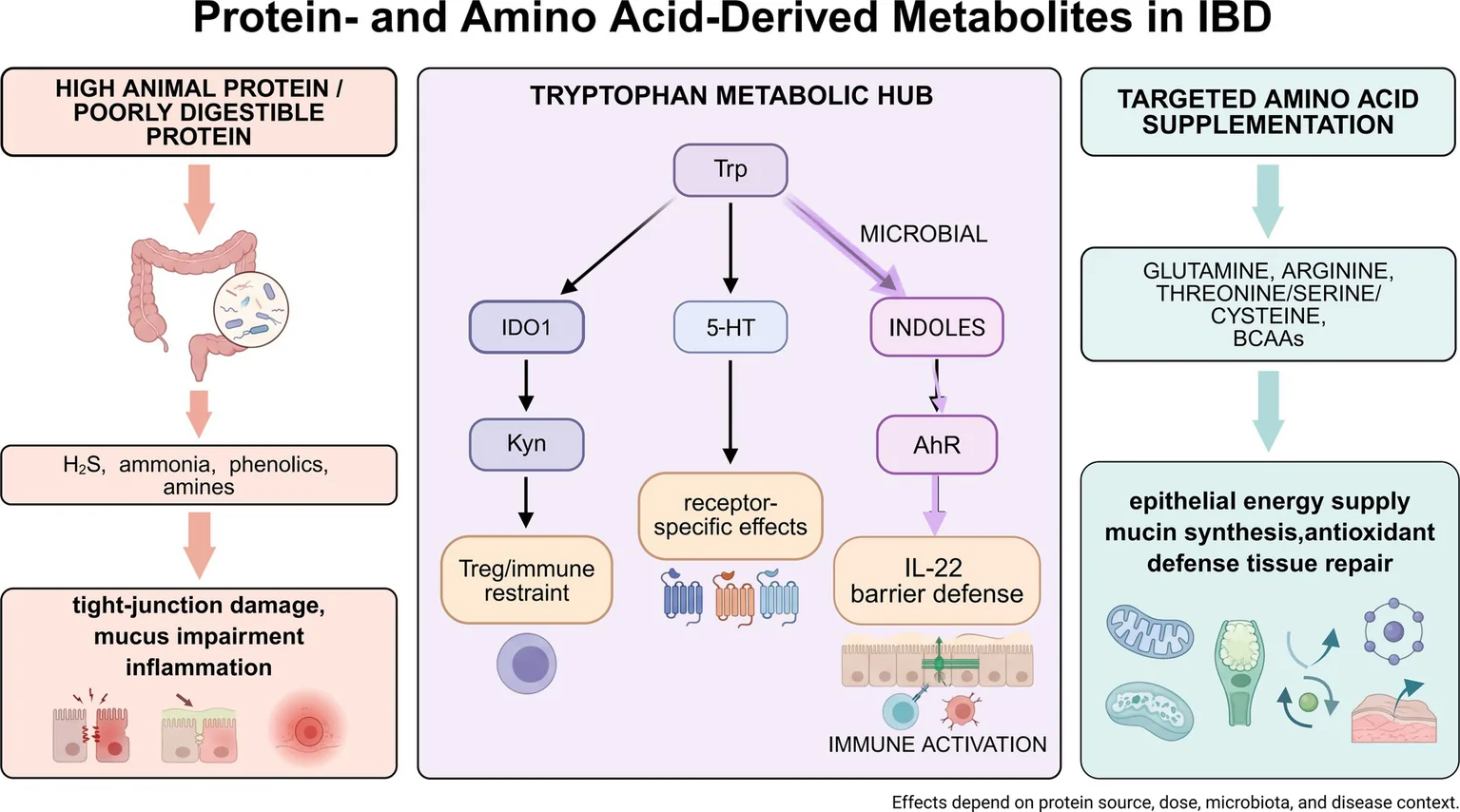
Protein-derived metabolites can exacerbate colonic inflammation, whereas specific amino acid pathways and targeted supplementation may support barrier repair in IBD. Colonic protein fermentation can generate hydrogen sulfide, ammonia, phenolic compounds, and amines that impair epithelial energy metabolism and barrier integrity. In contrast, host tryptophan metabolism through the IDO1–kynurenine and serotonin pathways, together with microbiota-derived indoles that activate AhR, regulates immune responses and epithelial homeostasis, including IL-22associated protective effects. Targeted amino acid supplementation may further support epithelial metabolism, mucus production, antioxidant defense, and mucosal repair. Created in BioRender. Yang, D. (2026) https://BioRender.com/kbkgpak.

Despite this extensive preclinical literature, the clinical translation of amino acid supplementation in IBD faces several unresolved challenges. First, most studies examine a single amino acid in isolation, whereas amino acids compete for transporters and their metabolism is interconnected—supplementing one may perturb the balance of others. Second, nearly all evidence derives from prevention models (supplementation initiated before or concurrently with colitis induction), which provides limited guidance for treating established disease. Third, the field lacks head-to-head comparisons and dose–response data, making it impossible to prioritize which amino acid, at which dose, for which patient, at which disease stage. Addressing these gaps will require not only well-designed clinical trials but also a deeper integration with the metabolic principles outlined earlier—specifically, how individual patients’ amino acid metabolic profiles (their Trp/Kyn ratio, arginine bioavailability, or gut microbial indole-producing capacity) might guide personalized supplementation strategies. The era of generic protein recommendations is giving way to a more nuanced paradigm in which amino acid supplementation, informed by metabolic phenotyping, could become a targeted component of precision nutrition for IBD (Figure 1).

## 3 Lipids and IBD

Two observations motivate an integrated examination of lipid metabolism in IBD. First, the intestinal epithelium itself is a lipid-dependent structure, and its integrity relies on a specific phospholipid composition (65). Second, the chronic inflammatory state of IBD rewires host lipid metabolism at every level—from local mediator production to systemic lipoprotein profiles. This lipid dysregulation is not merely a consequence of intestinal disease but actively participates in its pathogenesis.

A critical question is whether the lipid abnormalities observed in IBD are causes or consequences of intestinal inflammation. Recent Mendelian randomization studies have begun to clarify this relationship. While a 2024 bidirectional MR study found no causal evidence for conventional lipid indices (TC, TG, HDL-C, LDL-C) in IBD risk (66), a 2025 lipidome-wide MR analysis identified 14 lipid species—including phosphatidylcholine, lysophosphatidylcholine, and cholesterol esters—with significant causal associations with IBD, and reverse MR revealed no significant associations between IBD and 179 lipid species (67). Similarly, genetically predicted omega-3 fatty acids and DHA were shown to reduce IBD risk, with no reverse causal effect (68). These findings suggest that specific lipid species may play a causal role in IBD pathogenesis, whereas the broader reduction in TC, HDL-C, and LDL-C observed in IBD patients likely reflects a combination of inflammation-driven metabolic responses, malabsorption, and reduced dietary intake. The distinct lipid profiles observed in CD versus UC further argue against a single explanation and suggest that disease-specific inflammatory architectures produce distinct systemic metabolic signatures (69).

**Figure 2:**
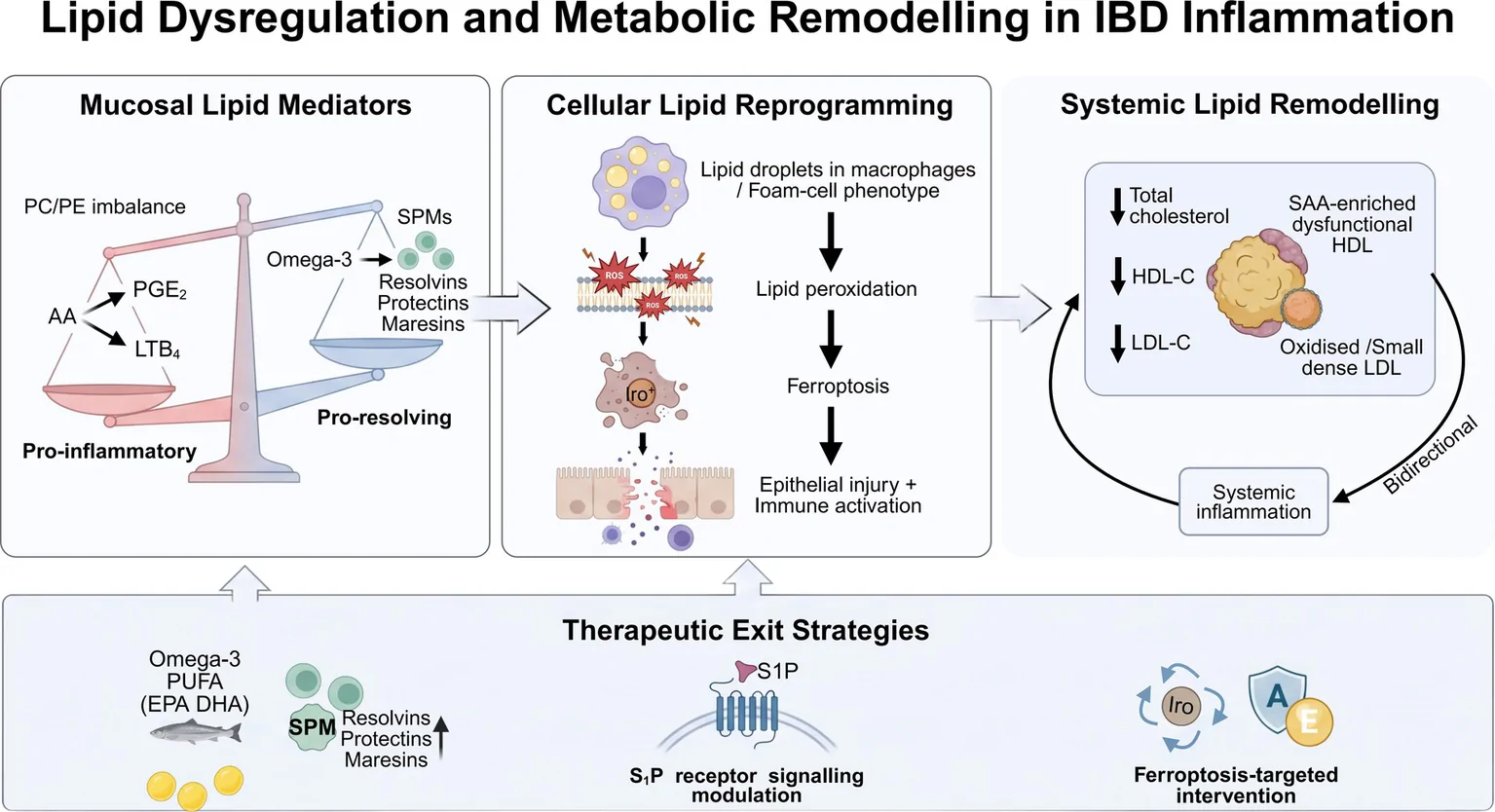
Lipid intake and lipid metabolic remodeling shape inflammatory responses and barrier integrity in IBD. At the mucosal level, altered membrane phospholipid composition increases the availability of pro-inflammatory lipid mediators, whereas inadequate omega-3-derived specialized pro-resolving mediators impairs inflammation resolution. At the cellular level, sphingolipid dysregulation, lipid droplet accumulation, lipid peroxidation, and ferroptosis contribute to epithelial injury and immune activation. Systemically, inflammation-associated lipoprotein remodeling, including serum amyloid A-enriched dysfunctional HDL, links intestinal inflammation with broader metabolic disturbances. Created in BioRender. Yang, D. (2026) https://BioRender.com/fezaefb.

This section was organized from the local to the systemic: beginning with the lipid composition of the epithelial barrier, moving through the pro-resolving versus pro-inflammatory lipid mediator network, then addressing immune cell lipid reprogramming and its systemic metabolic consequences, and finally considering the therapeutic implications of these interconnected processes (Figure 2).

### 3.1 Epithelial barrier lipid dependency and the lipid mediator network

The integrity of the intestinal epithelial barrier depends critically on the phospholipid composition of enterocyte membranes. In the inflamed mucosa of IBD patients, the ratio of phosphatidylcholine (PC) to phosphatidylethanolamine (PE) is significantly altered, and the lysophosphatidylcholine (LPC)/PC ratio correlates with the severity of inflammation (65). This phospholipid imbalance has functional consequences: membrane structural integrity declines, paracellular permeability increases, and bacterial antigens translocate into the lamina propria. The same phospholipid shift enriches arachidonic acid (ARA) while depleting linoleic acid and *α*-linolenic acid in membrane phospholipids (65), directly expanding the substrate pool for pro-inflammatory prostaglandins and leukotrienes at the expense of precursors required for SPMs. Thus, barrier failure and dysregulated lipid mediator production share a common phospholipid origin.

The resultant imbalance between pro-inflammatory and pro-resolving lipid mediators constitutes a defining feature of mucosal inflammation in IBD. On the pro-inflammatory side, AA-derived eicosanoids—prostaglandins (particularly PGE₂) and leukotrienes (notably LTB₄)—drive vascular permeability, leukocyte recruitment, and cytokine production, impairing epithelial regeneration and perpetuating inflammation (70–74). Counterbalancing this, SPMs derived from *ω*-3 fatty acids—including resolvins (RvE1, RvD1), protectins, and maresins (MaR1)—actively terminate inflammation by inhibiting neutrophil migration, promoting macrophage efferocytosis, and strengthening tight junction integrity (75–80). In IBD, systemic ω-3 fatty acid levels are reduced (81), and SPM production is inadequate relative to the magnitude of the inflammatory stimulus (82).

The sphingolipid network adds a further dimension to this mediator landscape. Ceramide, the central sphingolipid metabolite, is elevated in the inflamed mucosa of both UC and CD patients (83, 84), promoting NF-κB activation and apoptosis. Its phosphorylation product, sphingosine-1-phosphate (S1P), signals through five G protein-coupled receptors (S1PR1–5) to regulate immune cell trafficking and vascular integrity. S1PR1 is highly expressed in the colonic vasculature of UC patients, and its deficiency in mice impairs intestinal barrier function (85, 86). The activity of sphingomyelinases, particularly the protective alkaline sphingomyelinase (AlkSMase), is reduced in chronic colitis (87), further tilting the ceramide/S1P rheostat toward a pro-inflammatory state.

### 3.2 Immune cell lipid reprogramming and systemic lipid consequences

The inflammatory microenvironment of the intestine drives a metabolic reprogramming of infiltrating immune cells, in which lipid metabolism functions as a “molecular switch” (88). Inflammatory macrophages shift their metabolism away from fatty acid oxidation and toward fatty acid synthesis, resulting in the accumulation of lipid droplets. In the inflamed intestinal epithelium, lipid droplet accumulation is increased and depends on a FOXO3- and PGE₂-mediated lipogenic pathway (89). This is not a passive storage phenomenon; rather, it reflects a functional component of the inflammatory pathology, supplying substrates for eicosanoid synthesis and membrane biogenesis in activated immune cells.

A direct consequence of dysregulated lipid metabolism at the cellular level is ferroptosis—an iron-dependent form of regulated cell death driven by lipid peroxidation—which has emerged as a critical mechanism linking lipid metabolism to intestinal epithelial injury in IBD (90, 91). Key regulatory signaling networks implicated in this process include the Nrf2/HO-1, SLC7A11/GPX4, and AMPK/mTOR pathways (92). The microbiota–ferroptosis axis has also been implicated: *Fusobacterium nucleatum*, a periodontitis-associated pathobiont, exacerbates UC through ferroptosis-mediated gut barrier disruption (93).

Mitochondrial dysfunction is intimately linked to these processes; alterations in mitochondrial function within intestinal epithelial cells increase lipid peroxidation and promote ferroptosis (94, 95). Together, these findings extend the lipid-driven inflammatory cascade from mediator signaling to the direct oxidative consequences of dysregulated lipid metabolism on epithelial cell survival.

### 3.3 Systemic lipid abnormalities and causal interpretation

The local metabolic perturbations described above are reflected in—and compounded by—systemic lipid abnormalities. Meta-analyses consistently demonstrate reduced serum total cholesterol (TC), HDL cholesterol (HDL-C), and LDL cholesterol (LDL-C) in IBD patients, with further declines during active disease (69, 96, 97). The mechanisms are multifactorial and, importantly, differ between the lipoproteins. HDL-c reduction is driven by inflammation-induced hepatic synthesis of serum amyloid A (SAA), which displaces apolipoprotein AI on HDL particles, accelerating their catabolism and promoting HDL sequestration in adipose tissue (98–101). Because HDL facilitates cholesterol efflux from cell membranes and thereby depletes lipid rafts required for MHC class II surface expression, the fall in HDL-c impairs its immunoregulatory capacity—a self-reinforcing cycle in which inflammation lowers HDL and low HDL in turn compromises immune regulation (102). For LDL-C, inflammation-driven cholesteryl ester transfer protein activity enriches LDL with triglycerides; subsequent hydrolysis by hepatic lipase generates small, dense LDL particles that are more susceptible to oxidation and intrinsically more pro-inflammatory (103, 104). In parallel, TNF-*α* inhibits lipoprotein lipase (LPL) activity, yet serum LPL concentrations are paradoxically elevated in IBD (105), suggesting compensatory or inflammation-driven release. Apolipoprotein C-III (ApoC3) is downregulated in IBD patients (106), further affecting triglyceride metabolism.

A critical interpretative challenge lies in distinguishing whether these lipid alterations are causally involved in disease pathogenesis or merely epiphenomena of the inflammatory state. Reduced lipid levels in IBD patients may simply reflect malabsorption, reduced dietary intake, or active inflammation rather than direct pathogenic mechanisms (66–69). Conversely, the finding that SAA-enriched HDL loses its immunoregulatory capacity suggests that at least some lipid changes may actively contribute to disease perpetuation (102). The most parsimonious interpretation is bidirectional: inflammation drives lipid remodeling through hepatic acute-phase responses and impaired absorption, while the resulting lipid abnormalities—particularly dysfunctional HDL and pro-inflammatory LDL subspecies—feed back to amplify immune activation. Disentangling these causal pathways will require longitudinal studies with repeated measures during flares and remission, combined with Mendelian randomization approaches where feasible.

### 3.4 Therapeutic implications and future directions

The multi-level lipid dysregulation in IBD offers a correspondingly broad range of therapeutic opportunities, discussed in this study according to their site of action along the local–systemic continuum. The first and most intuitive strategy is dietary fatty acid modulation. Increasing *ω*-3 PUFA intake to shift the precursor balance toward SPM synthesis is supported by mechanistic plausibility and some clinical evidence, though results from large trials remain mixed, likely reflecting heterogeneity in baseline fatty acid status and genetic variation in fatty acid metabolism.

Pharmacological targeting of lipid signaling has gained considerable momentum through the sphingosine-1-phosphate receptor (S1PR) pathway, which represents the first class of lipid-pathway-targeting drugs approved for IBD. Ozanimod, a selective S1PR1 and S1PR5 modulator, demonstrated efficacy in the phase 3 TRUE NORTH trial, with long-term data showing durable responses (107). Etrasimod, a selective S1PR1, S1PR4, and S1PR5 modulator, showed significant efficacy in the ELEVATE UC 12 and 52 trials (108). A 2025 meta-analysis confirmed S1P receptor modulators as effective and safe treatments for UC (109).

In summary, the relationship between lipid metabolism and IBD operates on four interconnected levels: (i) at the molecular level, phospholipid membrane composition dictates barrier integrity and eicosanoid precursor availability; (ii) at the signaling level, the balance between pro-inflammatory mediators (prostaglandins, leukotrienes, ceramide) and SPMs determines whether inflammation resolves or persists; (iii) at the cellular level, immune cell metabolic reprogramming—particularly macrophage lipid droplet accumulation—is functionally embedded in the inflammatory process; and (iv) at the systemic level, altered lipoprotein metabolism reflects a complex interplay between inflammation, hepatic responses, and nutritional status. The bidirectional nature of these relationships—inflammation perturbing lipid metabolism, and lipid dysregulation amplifying inflammation—creates vicious cycles that are likely to sustain chronic disease. Breaking these cycles will require moving beyond descriptive lipidomic associations toward intervention studies that test whether specific lipid-targeted strategies—dietary, pharmacological, or microbial—can alter the trajectory of IBD in defined patient subsets.

## 4 Carbohydrates and IBD

Dietary carbohydrates are not a uniform entity. For understanding their relationship with IBD, the critical distinction lies between fermentable fiber—which serves as a substrate for SCFA production by the gut microbiota—and high-glycemic carbohydrates (refined sugars, fructose, and rapidly digestible starches) that are largely absorbed in the small intestine and, when consumed in excess, spill into the colon. Fiber deprivation and high sugar intake thus represent two sides of the same dietary pattern, and both have been independently linked to IBD risk. This section examines the three major mechanisms by which high-glycemic carbohydrates, in a fiber-poor context, contribute to intestinal inflammation: disruption of the gut microbial ecosystem, damage to the intestinal barrier, and direct metabolic reprogramming of colonic epithelial stem cells. These three levels are sequential in the microbiota-dependent pathway, yet the third also operates independently of the microbiota, forming a parallel route to mucosal injury.

### 4.1 Gut microbial ecosystem disruption (microbiota-dependent pathway, step 1)

The earliest consequence of a high-sugar, low-fiber diet is a shift in the gut microbial ecosystem. Two interrelated changes are particularly relevant to IBD pathogenesis.

The first is the expansion of mucolytic bacteria. Species such as *A. muciniphila* and *Bacteroides fragilis* possess enzymes that degrade the glycoproteins of the intestinal mucus layer. When dietary fiber is scarce, these bacteria increasingly rely on host mucins as an alternative energy source, and high sugar intake has been linked to their proliferation under certain conditions (110, 111). Recent studies have further demonstrated that sucrose-rich diets reshape the gut microbiota with marked blooms of *Enterococcus* (112), and that sugar-rich foods during antibiotic exposure are associated with expansion of the pathobiont *Enterococcus*, an observation validated experimentally in mice (113). Low dietary fiber, which frequently accompanies high-sugar diets, has also been shown to promote the enteric expansion of CD-associated pathobionts (114).

Collectively, these findings establish a direct link between carbohydrate availability—both the abundance of simple sugars and the absence of fermentable fiber—and the expansion of mucolytic and pathogenic taxa. More broadly, undigested carbohydrates reaching the colon promote the overgrowth of particular taxa at the expense of beneficial species such as *Lactobacillus* and *Bifidobacterium*, reducing both the diversity and the functional resilience of the gut ecosystem. The net result is a mucosal surface that is both more exposed—due to mucus thinning—and more vulnerable to colonization by organisms with pathogenic potential.

### 4.2 Barrier failure and mucosal immune activation (microbiota-dependent pathway, step 2)

The dysbiosis described above translates into intestinal barrier damage at multiple levels. The gut barrier depends on three integrated lines of defense: the mucus layer (physical barrier), tight junction proteins sealing the intercellular space (structural barrier), and the immunoregulatory milieu maintained by microbial metabolites (chemical barrier). A high-sugar, low-fiber diet weakens all three simultaneously.

First, the physical barrier is eroded: thinning of the mucus layer permits bacteria and their components—most notably lipopolysaccharide (LPS)—to contact the epithelial surface and, with sustained exposure, penetrate it. Second, the chemical barrier is depleted: fiber deprivation and the resulting dysbiosis reduce luminal SCFA concentrations. The three major SCFAs—acetate, propionate, and butyrate—are produced by bacterial fermentation of dietary fiber in the colon, with a typical molar ratio of approximately 60:20:20, respectively (115). Each contributes distinctively to intestinal homeostasis: butyrate serves as the primary fuel for colonocytes and promotes epithelial tight junctions; propionate supports regulatory T cell differentiation in the colonic lamina propria; and acetate modulates macrophage function through GPR43 signaling (115, 116). Importantly, meta-analysis data demonstrate significant reductions in fecal levels of all three SCFAs in IBD patients compared to healthy controls, with active IBD showing a greater decrease in butyrate (*p* = 0.004) and UC showing a notable reduction in propionate (*p* = 0.03) (116, 117). Transgenic mouse models have confirmed that GPR43 and GPR109A facilitate dietary fiber-induced gut homeostasis through inflammasome regulation (118). The deficiency of all three SCFAs—not merely butyrate—thus compromises both colonocyte energy supply and the immunoregulatory circuits that normally contain inflammatory responses. The resulting state is one of chronic, low-grade immune activation that persists as long as the barrier remains open and SCFA levels remain low.

### 4.3 Direct metabolic reprogramming of colonic epithelial stem cells

Alongside the microbiota-mediated effects described above, a microbiota-independent pathway has recently been defined through which high luminal glucose directly impairs the regenerative capacity of the colonic epithelium. Colonic epithelial cells express glucose transporters—most notably GLUT2 and SGLT1—that mediate the uptake of luminal glucose (119, 120). When colonic organoids are cultured under high-glucose conditions, glycolysis is enhanced but the tricarboxylic acid (TCA) cycle fails to keep pace. This creates a metabolic bottleneck: pyruvate accumulates, yet aerobic respiration does not correspondingly increase, leading to a net deficit in adenosine triphosphate (ATP) production (119). This energy shortfall directly suppresses the expression of genes required for stem cell proliferation. The functional importance of this bottleneck is demonstrated by a rescue experiment: when pyruvate is forced into the TCA cycle using dichloroacetate (DCA), organoid growth is restored, confirming that the metabolic defect lies at the glycolysis–TCA cycle interface (119). This mechanism is independent of the microbiota—it occurs in organoids cultured in the absence of bacteria—and therefore represents a parallel, direct route by which excess sugar can damage the gut.

Additional metabolic layers reinforce this vulnerability: pyruvate kinase M2 (PKM2), a rate-limiting glycolytic enzyme, is elevated up to 6-fold in the serum of IBD patients (121), yet this increased glycolytic flux does not translate into higher SCFA output because of the concurrent loss of SCFA-producing bacteria and fiber substrate (122). Similarly, alterations in O-GlcNAcylation, a post-translational modification that senses nutrient flux through the hexosamine pathway, are observed in the IBD epithelium and functionally contribute to barrier disruption (123).

It is important to acknowledge that the evidence for direct glucose-induced metabolic impairment of colonic stem cells derives primarily from organoid models and murine studies; the clinical relevance of this pathway in human IBD remains to be established. However, human data provide important context. GLUT2 and SGLT1 are expressed in the human colonic epithelium, as demonstrated by immunohistochemical analysis of colorectal mucosa from UC and CD patients (120), with no significant differences in transporter expression observed between IBD patients and healthy controls—suggesting that transporter presence is constitutive rather than disease-specific (120). Functional studies have shown that pro-inflammatory cytokines (IL-1β and TNF-*α*) upregulate SGLT1 gene expression, with SGLT1 regulation following the same pattern as TMPRSS2 under inflammatory conditions, implying a common regulatory pathway (124). Furthermore, studies in chronic intestinal inflammation models have demonstrated that SGLT1 transcription is regulated by Sp1 and HNF1 transcription factors, both of which are affected during inflammation (125). These observations suggest that while transporter expression per se may not be dramatically altered in IBD, the functional regulation of glucose transport and its metabolic consequences in the inflamed colonic epithelium warrant further investigation. Future studies employing metabolomic profiling of colonic biopsies and patient-derived organoids will be essential to validate the glycolysis–TCA uncoupling mechanism in the clinical setting.

### 4.4 Epidemiological evidence and a dual-pathway model

The mechanistic framework outlined above is supported by epidemiological data. Meta-analyses demonstrate that high intake of sugars—but not necessarily total carbohydrate intake—is positively associated with the risk of developing both UC (RR: 1.59; 95% CI: 1.15–2.20) and CD (RR: 1.90; 95% CI: 1.06–3.41) (126). The observation that total carbohydrate quantity and sugar-sweetened beverage consumption have not been consistently linked to IBD risk suggests that it is the specific type and metabolic handling of carbohydrates—rather than carbohydrate load as such—that constitutes the relevant pathogenic exposure.

**Figure 3:**
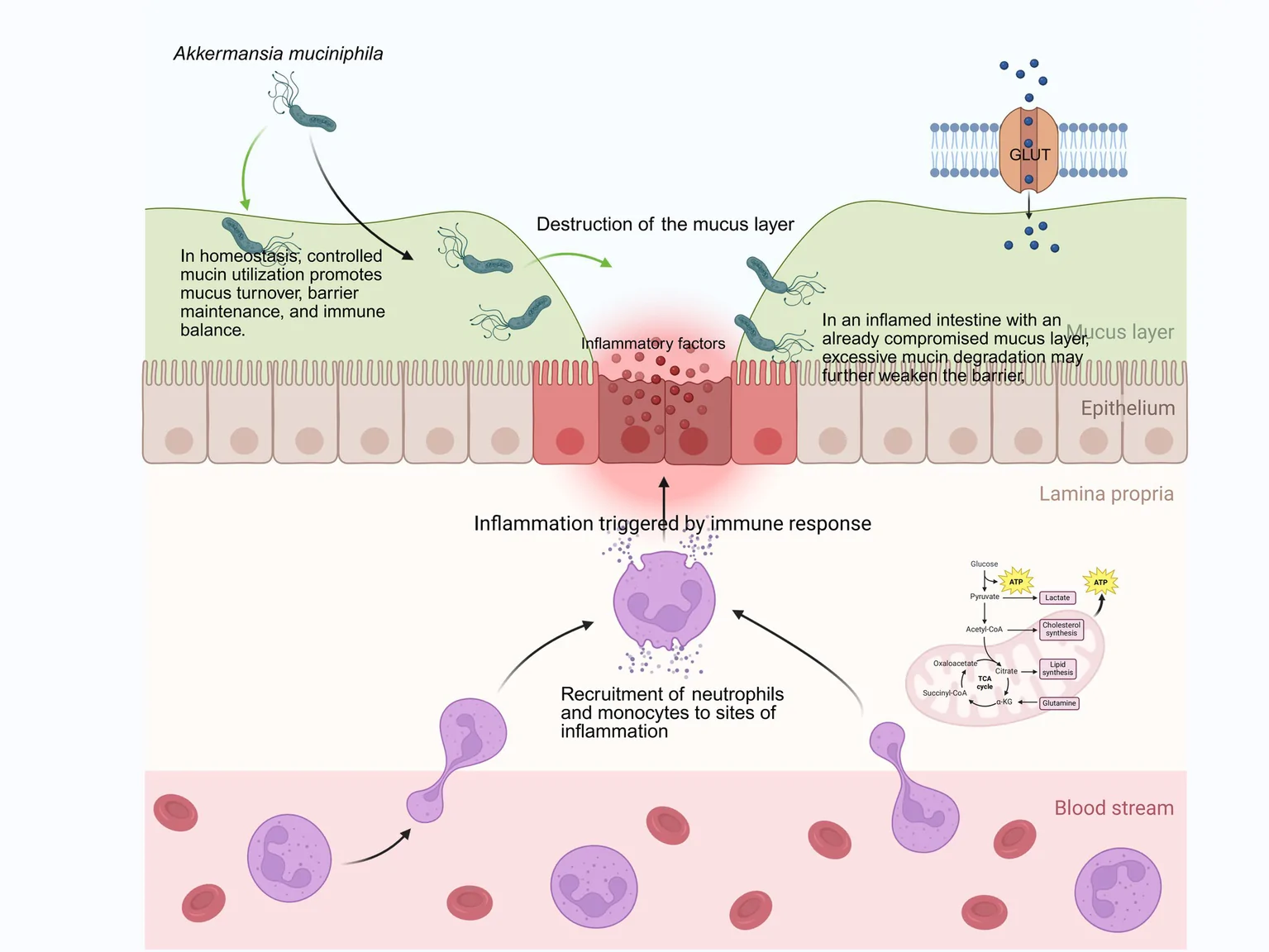
Excessive glucose intake disrupts mucus integrity and epithelial metabolism, promoting inflammatory responses in the colon. High glucose intake alters intestinal microbial activity and epithelial glucose handling through GLUT-mediated uptake, leading to metabolic reprogramming of epithelial cells. Enhanced glucose flux shifts cellular metabolism toward glycolysis and mitochondrial dysfunction, resulting in ATP deficiency and impaired epithelial renewal. Concurrently, dysregulated glucose availability compromises mucus layer integrity, increasing epithelial exposure to microbial products. Barrier disruption precedes immune activation, triggering recruitment of neutrophils and monocytes and amplifying inflammatory responses in the lamina propria. Together, glucose-driven metabolic imbalance links epithelial energy failure to barrier breakdown and chronic inflammation in the colon. Created in BioRender. Yang, D. (2026) https://BioRender.com/qnk1rx8.

Synthesizing the evidence, high-glycemic-carbohydrate diets are associated with IBD through two interacting but mechanistically distinguishable routes. The first is microbiota-dependent: low fiber and excess sugar, mucolytic and pathogenic bacterial expansion, mucus thinning → barrier breach, chronic immune activation fueled by reduced SCFA availability. The second is microbiota-independent: high luminal glucose, GLUT-mediated uptake, pyruvate accumulation and ATP deficit in colonic stem cells, impaired proliferative capacity, failed mucosal repair. These two pathways are not merely additive; they are synergistic. Barrier disruption increases the inflammatory load, which in turn further damages the epithelium and its stem cell compartment, while the energy deficit in stem cells prevents the resealing of the barrier that would normally terminate the inflammatory stimulus. Breaking this cycle is likely to require dietary strategies that address both sides simultaneously—reducing high-glycemic carbohydrate exposure while restoring fermentable fiber to support SCFA production, barrier integrity, and epithelial regeneration (Figure 3).

## 5 Discussion

This review has examined how dietary protein, lipids, and carbohydrates each influence the pathogenesis of IBD through distinct but interconnected metabolic routes. Rather than reiterating the individual pathways, emphasis is focused on three cross-cutting themes that emerge when these macronutrient effects are considered together, and on the translational challenges that must be addressed to move from mechanistic understanding to clinical application.

### 5.1 The limitations of single-nutrient research and the case for factorial designs

A recurring limitation of the literature surveyed here is its tendency to examine macronutrients in isolation. Yet the three metabolic networks described in this review do not operate independently. The gut microbiota sits at the intersection of all three: microbial composition is simultaneously shaped by dietary protein source (13, 14), by the ratio of fiber to high-glycemic carbohydrates, and by lipid intake (110, 111, 127). A shift in one dietary component inevitably alters the microbial context in which the others are metabolized. For example, the expansion of mucolytic bacteria under high-sugar, low-fiber conditions (112, 113)would be predicted not only to deplete SCFAs but also to alter the microbial taxa responsible for tryptophan-to-indole conversion; the consequent reduction in AhR ligand availability would compound the barrier defect caused by butyrate deficiency. This raises the experimentally testable hypothesis that the deleterious effects of a high-sugar diet on colitis severity could be partially mitigated by co-administration of tryptophan or AhR-activating probiotics (42–45). Similarly, the systemic lipid profile described in Section 3.2 is unlikely to be diet-independent: the fatty acid composition of the diet directly influences the substrate available for SPM synthesis, and thus the host’s capacity to resolve inflammation in the face of ongoing barrier damage (75–80). The field urgently needs factorial dietary intervention studies in animal models—and, where feasible, in humans—that manipulate two macronutrients simultaneously, specifically testing for synergistic or antagonistic interactions. Without such data, the common practice of extrapolating from single-nutrient studies to complex dietary advice rests on an uncertain foundation.

### 5.2 Multi-omics integration as a strategy to move beyond single-nutrient studies

The recognition that single-nutrient studies cannot capture the complexity of diet–host–microbe interactions points toward multi-omics integration as a strategy to move beyond these limitations. Recent multi-omics studies have begun to identify specific microbial and metabolic signatures that distinguish IBD patients from healthy controls. For example, integrated metagenomic and metabolomic analyses have revealed that IBD is characterized by depletion of *Faecalibacterium prausnitzii* and reduced butyrate production, alongside elevated amino acid fermentation products and altered bile acid profiles, with distinct signatures for CD and UC (128). In the context of dietary intervention, multi-omics approaches have shown that exclusive enteral nutrition remodels the gut microbiome and metabolome in ways that correlate with clinical remission, providing mechanistic insights beyond simple nutrient provision (129). Metabolomic profiling has further identified plasma metabolites—including tryptophan metabolites, sphingolipids, and acylcarnitines—that correlate with disease activity and may serve as biomarkers for treatment response (130). From a nutrigenomic perspective, polymorphisms in genes encoding fatty acid desaturases (FADS1/2) influence the conversion of dietary *α*-linolenic acid to EPA and DHA, potentially explaining inter-individual variability in response to *ω*-3 supplementation (131), while genetic variation in the aromatic amino acid decarboxylase (DDC) gene may influence tryptophan metabolism and AhR signaling (132), These examples illustrate how multi-omics approaches are beginning to generate the data needed to identify biomarkers for guiding nutritional interventions, though prospective validation remains a critical next step.

### 5.3 Convergent mechanisms: the barrier–metabolism–immune triangle

Despite their distinct entry points, the three macronutrient pathways converge on a shared functional triangle: intestinal barrier integrity, epithelial energy metabolism, and mucosal immune regulation. In the protein and amino acid domain, tryptophan metabolites—kynurenines, serotonin, and indole derivatives—simultaneously affect tight junction proteins, epithelial cell proliferation, and cytokine profiles (21, 29, 41). Arginine metabolism, through the iNOS–arginase balance, determines whether the local milieu tilts toward inflammatory tissue injury or reparative polyamine synthesis (Section 2.2). In the lipid domain, the phospholipid composition of the enterocyte membrane dictates the physical properties of the barrier while also determining the precursor pools for pro- and anti-inflammatory eicosanoids (65). In the carbohydrate domain, the distinction between microbiota-dependent barrier disruption (Section 4.2) and microbiota-independent stem cell metabolic impairment (Section 4.3) illustrates how a single dietary exposure can damage the same functional endpoint—epithelial integrity—through mechanistically independent routes that are likely additive *in vivo*. This convergence implies that successful nutritional therapy for IBD will probably need to address multiple nodes simultaneously. It also suggests that composite biomarkers—for instance, a panel that includes the Kyn/Trp ratio (21), a specific SPM profile (81, 82), and a measure of epithelial proliferative capacity—may prove more informative than any single metabolite measured in isolation.

### 5.4 Remaining controversies and knowledge gaps

Several unresolved questions merit emphasis. First, the causal direction of many diet–microbiome–inflammation associations remains undetermined. The consistent observation that IBD patients show reduced serum TC, HDL-C, and LDL-C, with further declines during active disease (69, 96, 97), illustrates this difficulty: are these reductions a consequence of inflammation, malnutrition, and malabsorption, or do they actively contribute to disease perpetuation? The finding that SAA-enriched HDL loses its immunoregulatory capacity (102) supports a functional link, but longitudinal studies with repeated measures during disease flares and remission, combined with Mendelian randomization where feasible, would be required to disentangle cause from effect. Second, the epidemiological data present their own interpretive challenges. The association between total meat intake and increased IBD risk highlights how the food matrix—the combination of nutrients, fermentation products, and bioactive compounds in a whole food—confounds attempts to attribute risk to a single macronutrient (133, 134). Disentangling these effects requires moving beyond food frequency questionnaires to studies that use controlled diets with matched macronutrient composition that differ in specific food sources. Third, the dose–response and temporal dimensions of nutrient effects are almost entirely unexplored. Most amino acid supplementation studies have used prevention models in which supplementation is initiated before or concurrently with colitis induction (54, 57–64, 135), providing limited guidance for patients with established disease. Determining whether a given nutrient intervention is most effective as a preventive strategy, an acute therapy during flares, or a maintenance agent during remission will require studies with timed interventions at distinct disease phases. Fourth, CD and UC exhibit different dietary risk associations and lipid profiles (69), the IOIBD’s differential recommendations for meat intake in CD versus UC are a case in point (134, 136)—yet the metabolic basis for these differences remains unknown.

### 5.5 Toward a framework for metabolic stratification and clinical translation

To translate these mechanistic insights into clinically actionable strategies, a preliminary framework for metabolic stratification of IBD patients was proposed based on three complementary domains—amino acid, lipid, and carbohydrate metabolism—each of which is directly supported by the evidence reviewed in this article.

In the amino acid domain, patients could be stratified by their plasma tryptophan–kynurenine ratio (Kyn/Trp), which correlates with IDO1 activity and disease activity in IBD, and by their gut microbial indole-producing capacity, which determines the availability of AhR ligands essential for IL-22-mediated epithelial protection. Patients with elevated Kyn/Trp ratios or depleted indole pathway activity might be prioritized for tryptophan supplementation or AhR-targeting postbiotic interventions, whereas those with preserved indole metabolism may require different nutritional support.

In the lipid domain, stratification could be based on the plasma *ω*-6/ω-3 polyunsaturated fatty acid ratio and the levels of specialized pro-resolving mediators (SPMs), which together reflect the balance between pro-inflammatory and pro-resolving signaling in the mucosa. Patients with a high ω-6/ω-3 ratio and depleted SPMs might benefit from ω-3 PUFA enrichment or SPM-mimetic strategies, while those with a more balanced profile may not require such interventions. The SAA-enriched HDL phenotype, which reflects inflammation-driven HDL dysfunction and loss of immunoregulatory capacity, could further refine stratification by identifying patients in whom lipid-targeted therapies may be most beneficial.

In the carbohydrate domain, stratification could be guided by fasting glucose levels, serum PKM2 concentrations, and fecal SCFA profiles. Patients with elevated PKM2 and reduced fecal SCFAs—reflecting the glycolysis–TCA cycle bottleneck in colonic epithelial stem cells and concurrent SCFA depletion—may be most likely to benefit from dietary sugar reduction and fermentable fiber restoration, as these interventions directly address the dual-pathway model of carbohydrate-driven injury described in Section 4.

This three-domain framework, while requiring prospective validation in well-designed cohort studies and randomized trials, offers a testable approach to matching nutritional strategies to individual metabolic phenotypes. Its implementation currently faces practical challenges, including the need for standardized assays, validated cut-off values, and integration with routine clinical workflows. Nevertheless, the study provides a conceptually grounded starting point for moving beyond generic dietary advice toward mechanism-informed and personalized nutritional strategies in IBD.

### 5.6 Prioritizing strategies for clinical evaluation

Translating the mechanistic insights reviewed here into interventions that can be tested in clinical trials requires prioritizing the strategies with the most favorable feasibility-to-evidence ratio. Based on the weight of evidence summarized in this review, three approaches appear particularly ready for clinical evaluation.

First, adequate protein and specific amino acid provision during active disease, as already endorsed by ESPEN guidelines (1.2–1.5 g/kg/day) (51), deserves prospective testing with endpoints that include not only nutritional status but also mucosal healing and inflammatory biomarkers. Given the preclinical data on glutamine (52, 54–56, 135) and arginine (61–63), trials comparing standard polymeric formulas with formulas enriched in these amino acids during exclusive enteral nutrition for CD would be a logical next step. Second, dietary strategies that simultaneously reduce high-glycemic carbohydrate intake and ensure adequate fermentable fiber—rather than manipulating either variable in isolation—are strongly supported by the dual-pathway model described in Sections 4.2 and 4.3 and warrant evaluation for maintenance of remission. The epidemiological evidence that sugar type, rather than total carbohydrate quantity, is associated with IBD risk (126) further supports a qualitative rather than purely quantitative approach to carbohydrate guidance. Third, the sphingolipid axis is the only pathway discussed here for which a clinically approved therapy already exists—S1PR modulators for UC (108)—validating the translational potential of targeting lipid signaling pathways; studying the metabolic effects of existing nutritional interventions on this axis could identify dietary strategies that achieve similar immunomodulatory outcomes with a more favorable safety profile.

## 6 Conclusion

In conclusion, the relationship between dietary macronutrients and IBD is bidirectional, multi-layered, and operates through mechanisms that extend well beyond simple caloric supply. The evidence synthesized in this review supports a shift away from generic dietary advice and toward a more nuanced, mechanism-informed approach in which the metabolic fates of specific amino acids, fatty acids, and carbohydrates are considered as modifiable determinants of mucosal immune homeostasis. The challenge for the next generation of intervention studies is to determine whether this approach—matching nutritional strategies to individual metabolic phenotypes and disease stages—can improve clinical outcomes above and beyond current standard nutritional care. To facilitate cross comparison and provide a concise integrative overview of the core findings across the three macronutrient classes, the principal mechanisms, microbial and barrier effects, immune outcomes, and key mediators are summarized in Tables 2, 3.

**Table 2:** Key metabolic pathways and key mediators of dietary macronutrients in IBD.

Macronutrient	Key metabolic pathways	Key mediators
Protein	Tryptophan (IDO-Kyn/5-HT/AhR), Arginine (iNOS/Arginase)	Kyn/Trp ratio, AhR ligands, NONitric oxideNO, Polyamines
Lipid	Phospholipid remodeling (PC/PE), Eicosanoid/SPM synthesis, Ceramide/S1P signaling	AA/EPA/DHA ratio, PGE₂, LTB₄, Resolvins, Ceramide/S1P, HDL-c, sdLDL-c
Carbohydrate	Glycolysis-TCA uncoupling, SCFA(acetate/propionate/butyrate) oxidation, O-GlcNAcylation	Butyrate, acetate, propionate, LPS, PKM2, ATP, O-GlcNAc, GLUT2/SGLT1

**Table 3:** Effects on gut microbiota, epithelial barrier, and mucosal immunity of dietary macronutrients in IBD.

Macronutrient	Effects on Gut microbiota	Effects on epithelial barrier
Protein	Protein fermentation increases H₂S, phenols, and ammonia; animal protein enriches mucolytic/pro-inflammatory taxa (*Akkermansia*, *Enterococcus*, *Streptococcus*)	Amino acids (Thr, Ser, Cys) support mucin synthesis; H₂S/ammonia compromise tight junctions; AhR ligands promote epithelial repair
Lipid	Dietary ω-3/ω-6 ratio influences SCFA-producing taxa; probiotics modulate host lipid metabolism	PC/PE imbalance increases membrane permeability; SPMs (RvE1, MaR1) tighten junctions; ceramide promotes epithelial apoptosis
Carbohydrate	High sugar expands mucolytic bacteria; low fiber reduces SCFA producers (*Faecalibacterium*, *Roseburia*)	Mucus thinning; SCFA deficiency impairs tight junctions; GLUT-mediated glucose uptake reduces stem cell ATP and proliferation
